# Clinical Outcomes of Second-Line Amrubicin and Platinum-Based Doublet Chemotherapy in Patients with Extrapulmonary Neuroendocrine Carcinoma: A Multicenter Retrospective Study

**DOI:** 10.3390/cancers18162664

**Published:** 2026-08-18

**Authors:** Nana Yamakita, Taiki Okumura, Saki Ota, Shoichiro Yoneyama, Takuro Noguchi, Yasushi Sekino, Masato Nakamura, Akihiro Shinji, Nobumichi Takeuchi, Shintaro Kanda

**Affiliations:** 1 Shinshu University School of Medicine, Matsumoto 390-8621, Nagano, Japan; 21m0111a@shinshu-u.ac.jp; 2 Shinshu Cancer Center, Shinshu University Hospital, Matsumoto 390-8621, Nagano, Japan; taknoguchi@shinshu-u.ac.jp (T.N.); skanda@shinshu-u.ac.jp (S.K.); 3 Department of Medicine, Division of Gastroenterology and Hepatology, Shinshu University School of Medicine, Matsumoto 390-8621, Nagano, Japan; 4 Department of Pharmacy, Shinshu University Hospital, Matsumoto 390-8621, Nagano, Japan; ohta@shinshu-u.ac.jp; 5 Department of Gastroenterology, Suwa Red Cross Hospital, Suwa 392-8510, Nagano, Japan; shoichiroyoneyama1995@outlook.jp; 6 Department of Gastrointestinal Surgery, Nagano Municipal Hospital, Nagano 381-8551, Nagano, Japan; yasushi_sekino@hospital.nagano.nagano.jp; 7 Aizawa Comprehensive Cancer Center, Aizawa Hospital, Matsumoto 390-0814, Nagano, Japan; geka-dr7@ai-hosp.or.jp; 8 Department of Medical Oncology, Suwa Red Cross Hospital, Suwa 392-8510, Nagano, Japan; smile-shinji@po32.lcv.ne.jp; 9 Department of Medical Oncology, Ina Central Hospital, Ina 396-0033, Nagano, Japan; ntakeuti@inahp.jp

**Keywords:** amrubicin, platinum-doublet chemotherapy, neuroendocrine carcinoma

## Abstract

**Background/Objectives**: Extrapulmonary neuroendocrine carcinoma (EPNEC) is a rare, heterogeneous malignancy with no standard second-line treatment after progression on first-line platinum-doublet chemotherapy. We compared the efficacy and safety of amrubicin (AMR) versus platinum-based doublet as second-line therapy for advanced EPNEC. **Methods**: This multicenter retrospective study included patients with advanced EPNEC treated at five Japanese institutions who received second-line AMR or a platinum-based doublet. Overall survival (OS), progression-free survival (PFS), objective response rate (ORR), disease control rate (DCR), and adverse events were assessed. **Results**: Forty-four patients were enrolled (platinum-based doublet, *n* = 25; AMR, *n* = 19). Median OS was 6.1 months (95% confidence interval [CI], 4.9–9.0) and 6.8 months (95% CI, 2.7–13.2), and median PFS was 2.2 months (95% CI, 1.6–4.4) and 2.1 months (95% CI, 1.5–6.8), respectively. ORR and DCR were 20.0% and 32.0% in the platinum group and 5.3% and 26.3% in the AMR group, respectively. No significant differences were observed for OS, PFS, ORR, or DCR. Grade 3–4 hematologic toxicity was more frequent in the platinum group, whereas severe non-hematologic toxicity was rare in both groups. **Conclusions**: AMR therapy and platinum-based doublet therapy may exhibit comparable efficacy and acceptable safety profiles as second-line treatment options for advanced EPNEC.

## Simple Summary

Extrapulmonary neuroendocrine carcinoma (EPNEC) is a rare and aggressive cancer with no established standard second-line treatment after failure of first-line platinum-based chemotherapy. We retrospectively compared the efficacy and safety of amrubicin (AMR) and platinum-based doublet chemotherapy in 44 patients with advanced EPNEC treated at five Japanese institutions. Median overall survival was 6.1 months with platinum-based doublet therapy and 6.8 months with AMR, while median progression-free survival was 2.2 and 2.1 months, respectively. Objective response and disease control rates were also similar between the two groups, with no statistically significant differences in efficacy outcomes. Severe hematologic adverse events occurred more frequently with platinum-based doublet therapy, whereas serious non-hematologic toxicities were uncommon in both groups. These findings suggest that AMR and platinum-based doublet chemotherapy may provide comparable clinical benefit with manageable safety, supporting both as reasonable second-line treatment options for patients with advanced EPNEC.

## 1. Introduction

Extrapulmonary neuroendocrine carcinoma (EPNEC) is a rare and highly aggressive malignancy with a poor prognosis [1,2,3]. Treatment remains challenging because of marked pathological heterogeneity, including differences in primary site, histological characteristics, proliferative activity, and molecular profiles [4,5]. Although neuroendocrine carcinoma (NEC) can arise in various organs, most patients with advanced or recurrent disease require systemic chemotherapy, and long-term outcomes remain unsatisfactory [6,7]. Systemic chemotherapy for EPNEC is generally guided by treatment strategies established for small-cell lung cancer (SCLC). Platinum-based doublet chemotherapy with etoposide or irinotecan has been established as the standard first-line treatment for advanced NEC, based on prospective clinical studies and accumulated clinical evidence [8,9]. Nonetheless, despite initial responses to platinum-based therapy, most patients eventually experience disease progression and require subsequent treatment. In contrast to the first-line setting, no standard second-line chemotherapy is currently established for patients with advanced EPNEC. Several cytotoxic agents and combination regimens have been used in clinical practice, but the available evidence is limited, and treatment recommendations are largely based on retrospective analyses and institutional experience [10,11]. Consequently, management after failure of platinum-based chemotherapy remains heterogeneous. Amrubicin, a fully synthetic anthracycline and potent topoisomerase II inhibitor, has demonstrated efficacy in relapsed small-cell lung cancer and was subsequently investigated in patients with previously treated NEC. Several small retrospective studies have suggested that amrubicin may provide clinically meaningful antitumor activity with an acceptable safety profile [12,13,14]. However, these reports were limited by small sample sizes and single-center designs, and the role of amrubicin in NEC remains unclear. Platinum-doublet chemotherapy has also been used as a second-line treatment option in selected patients, particularly those who achieved favorable responses to prior platinum-based therapy [15]. Evidence supporting this strategy is limited, and no study has directly compared the effectiveness of amrubicin and platinum-doublet chemotherapy in the second-line setting [16]. Therefore, we conducted a multicenter retrospective study to compare the efficacy and safety of amrubicin and platinum-doublet chemotherapy as second-line treatment for advanced EPNEC.

## 2. Materials and Methods

### 2.1. Patients

This multicenter retrospective study included consecutive patients with EPNEC who received second-line chemotherapy after failure of first-line platinum-based chemotherapy at five institutions in Nagano Prefecture, Japan (Shinshu University Hospital, Suwa Red Cross Hospital, Nagano Municipal Hospital, Aizawa Hospital, and Ina Central Hospital), between January 2013 and June 2026. The diagnosis of NEC was established histologically according to the 2022 World Health Organization classification of endocrine and neuroendocrine tumors (5th edition) [17,18,19]. Histopathological findings included poorly differentiated neuroendocrine morphology with small-cell or large-cell features. Immunohistochemical analyses were performed using neuroendocrine markers, including chromogranin A, synaptophysin, and/or CD56, to confirm the diagnosis [17,18,19,20]. Clinical, pathological, treatment, and survival data were retrospectively extracted from medical records at each participating institution. Patients who received either amrubicin monotherapy or platinum-doublet chemotherapy as second-line treatment were eligible for inclusion in this analysis. The selection of second-line treatment was left to the discretion of the treating physician, as no standardized treatment selection criteria were established across participating institutions. In clinical practice, treatment selection may have been influenced by patient characteristics, prior treatment response and tolerability, disease status, patient preference, and institutional or physician preferences. Patients received one of the following regimens at the discretion of the treating investigator: cisplatin plus irinotecan (CDDP + IRI), consisting of irinotecan (60 mg/m^2^ on days 1, 8, and 15) and cisplatin (60 mg/m^2^ on day 1), repeated every 4 weeks; carboplatin plus etoposide (CBDCA + ETP), consisting of etoposide (80–100 mg/m^2^ on days 1, 2, and 3) and carboplatin (area under the curve [AUC] 4–5 on day 1), repeated every 3 weeks; carboplatin plus paclitaxel (CBDCA + PTX), consisting of paclitaxel (200 mg/m^2^ on day 1) and carboplatin (AUC 6 on day 1), repeated every 3 weeks; carboplatin plus irinotecan (CBDCA + IRI), consisting of irinotecan (60 mg/m^2^ on days 1, 8, and 15) and carboplatin (AUC 4–5 on day 1), repeated every 4 weeks; or amrubicin (AMR) monotherapy (35–45 mg/m^2^ on days 1, 2, and 3), repeated every 3 weeks. Dose modifications and treatment delays were permitted according to the investigator’s clinical judgment and institutional practice. The use of granulocyte colony-stimulating factor (G-CSF), including primary and secondary prophylaxis, was considered at the discretion of the physician based on individual patient factors. Follow-up began at the initiation of second-line chemotherapy and ended at the last visit or death. Tumor response was assessed by CT or MRI 6–12 weeks after treatment initiation and approximately every 2 months thereafter, using standardized assessment criteria based on RECIST v1.1 across all participating institutions [21]. The objective response rate (ORR) was defined as the proportion of patients with complete response (CR) or partial response (PR), and disease control rate (DCR) as the proportion with CR, PR, or stable disease at the first assessment. ORR and DCR were calculated in the full analysis set, and patients without any post-baseline tumor assessment were considered non-responders and as not having achieved disease control, respectively.

### 2.2. Staging

As no established staging system exists for EPNEC, disease stage was classified based on distant metastasis: tumors without distant metastasis were classified as “locally advanced,” whereas those with distant metastasis were classified as “metastatic” [9,22].

### 2.3. Adverse Events

Adverse events (AEs) were graded according to the National Cancer Institute Common Terminology Criteria for Adverse Events, version 5.0.

### 2.4. Ethics

This multicenter retrospective study was approved by the Institutional Review Board of Shinshu University School of Medicine (approval number: 6716), which served as the central ethics committee. The study was conducted in accordance with the principles of the Declaration of Helsinki (as revised in 2013) and the Ethical Guidelines for Medical and Health Research Involving Human Subjects in Japan. Written informed consent was waived due to the retrospective study design. Instead, an opt-out approach was adopted. Information regarding the study was publicly disclosed on the website of Shinshu University School of Medicine, and patients were provided with the opportunity to decline participation (https://www.shinshu-u.ac.jp/faculty/medicine/chair/i-hemon/). All clinical management and treatments were performed after obtaining appropriate informed consent for routine medical care from each patient.

### 2.5. Statistical Analysis

Statistical analyses and data visualization were performed using StatFlex ver. 7.0.11 (Artech Co., Ltd., Osaka, Japan). Baseline characteristics were assessed before second-line chemotherapy and summarized as medians (interquartile ranges) for continuous variables and frequencies (percentages) for categorical variables. Continuous and categorical variables were compared using the Mann–Whitney U test and chi-square or Fisher’s exact test, respectively. Overall survival (OS) was defined as the time from second-line chemotherapy initiation to death, and progression-free survival (PFS) as the time to disease progression or death. OS and PFS were estimated using the Kaplan–Meier method and compared with the log-rank test. All tests were two-sided, with *p* < 0.05 considered statistically significant.

## 3. Results

### 3.1. Patient Characteristics

**Figure 1:**
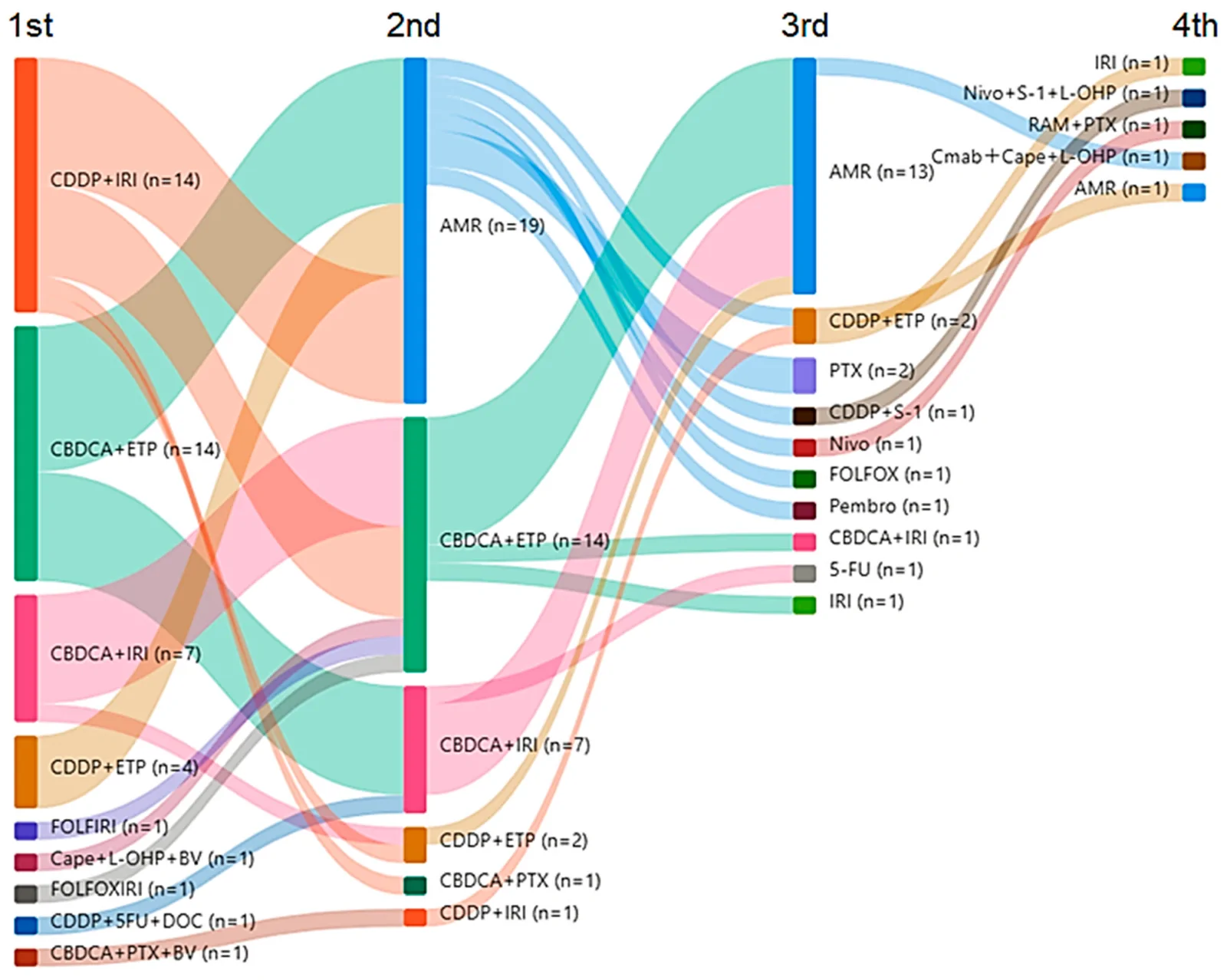
Treatment sequences across systemic therapy lines in patients with advanced extrapulmonary neuroendocrine carcinoma (EPNEC). Diagram illustrating the transition of systemic treatment regimens from first-line (1st) to fourth-line (4th) therapy. The width of each flow is proportional to the number of patients receiving each treatment sequence. Numbers in parentheses indicate the number of patients treated with each regimen at each treatment line.

Overall, 44 patients were included in this study: 25 received platinum-based second-line chemotherapy, and 19 received second-line AMR. The median follow-up duration was 12.5 months (95% CI, 8.6–16.4 months), as estimated using the reverse Kaplan–Meier method. Table 1 summarizes patient characteristics. In the platinum group, 20 patients (80.0%) were male. The most common primary tumor site was the gastrointestinal tract (56.0%), followed by the hepatobiliary and pancreatic system (20.0%). In the AMR group, 13 patients (68.4%) were male. Similarly, the gastrointestinal tract was the most common primary tumor site (63.2%), followed by the hepatobiliary and pancreatic system (15.8%). Metastatic disease was present in most patients in both the platinum group (92.0%) and the AMR group (78.9%). In the platinum group, ECOG performance status (PS) was 0, 1, and 2 in five (20.0%), 16 (64.0%), and four (16.0%) patients, respectively. The corresponding numbers in the AMR group were seven (36.8%), eight (42.1%), and three (15.8%). No significant difference was observed between the groups (*p* = 0.563). The median number of second-line treatment cycles was 2.0 (interquartile range [IQR]: 1.0–4.3) in the platinum group and 3.0 (IQR: 2.0–4.8) in the AMR group. In the platinum group (*n* = 25), primary prophylactic G-CSF, secondary prophylactic G-CSF, and no G-CSF administration were reported in five (20.0%), 10 (40.0%), and 10 (40.0%) patients, respectively. In the AMR group (*n* = 19), the corresponding counts were 13 (68.4%), one (5.3%), and four (21.1%), respectively, and G-CSF use was unknown for one patient. Treatment sequences are illustrated in Figure 1. Platinum-based chemotherapy was the predominant first-line treatment and consisted mainly of CDDP + IRI and CBDCA + ETP. In the second-line setting, AMR was the most frequently administered regimen (*n* = 19). The second most frequently administered regimen was CBDCA + ETP (*n* = 14), which tended to be selected as second-line treatment for patients who had not received ETP-containing chemotherapy in the first-line setting. Beyond second-line treatment, therapeutic strategies became increasingly heterogeneous, with patients receiving a variety of subsequent systemic therapies, including platinum rechallenge, taxane-based regimens, fluoropyrimidine-based chemotherapy, and immune checkpoint inhibitors. Overall, 24 patients (54.5%) received third-line therapy, and five patients (11.4%) received fourth-line therapy.

### 3.2. Efficacy

**Figure 2:**
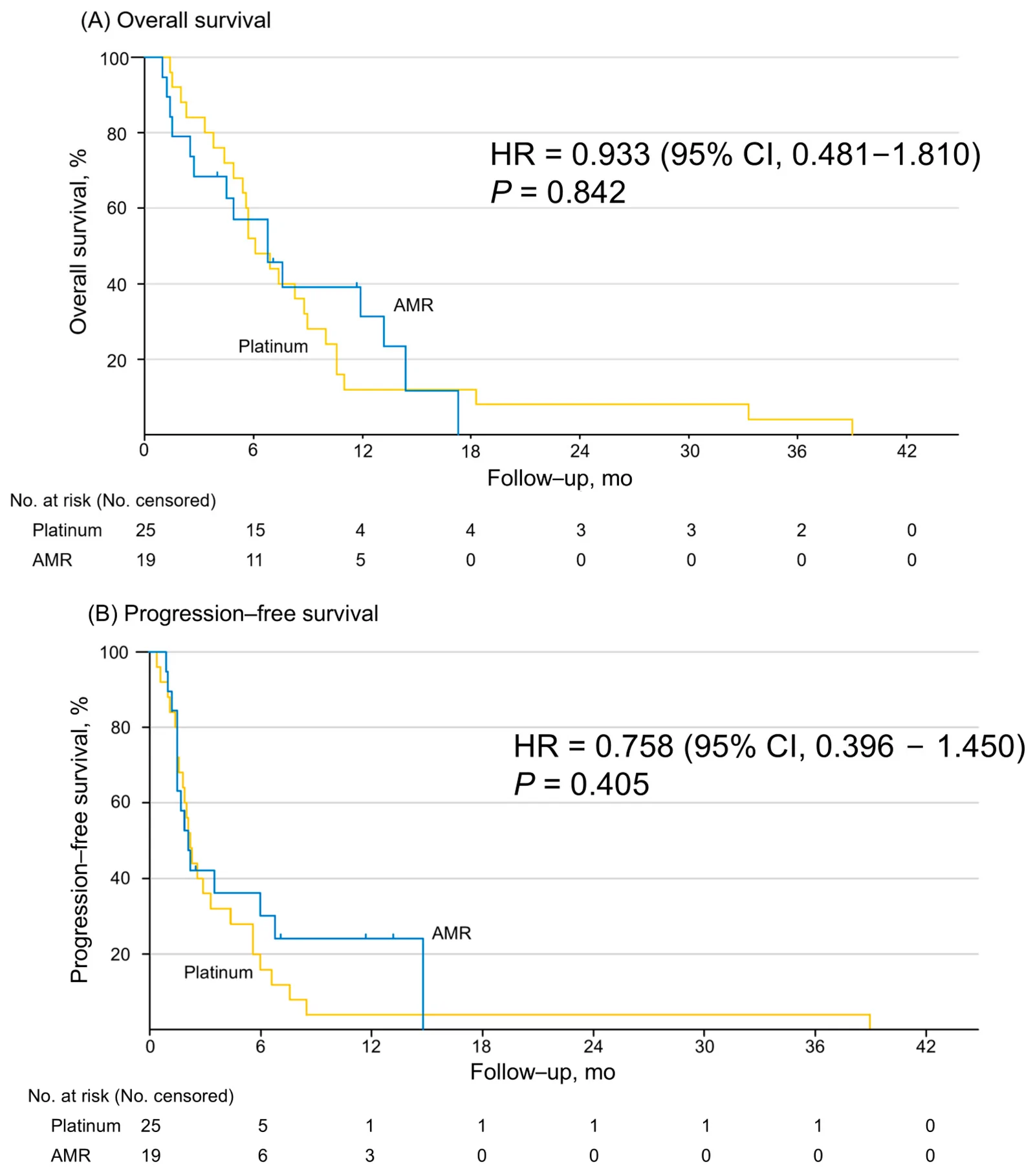
Kaplan–Meier curves for overall survival and progression–free survival according to second-line treatment. (**A**) Overall survival (OS) and (**B**) progression-free survival (PFS) in patients with advanced extrapulmonary neuroendocrine carcinoma (EPNEC) who received second-line amrubicin (AMR) or platinum-based doublet chemotherapy. Tick marks indicate censored observations.

The median OS in the platinum and AMR groups was 6.1 months (95% confidence interval [CI]: 4.9–9.0) and 6.8 months (95% CI: 2.7–13.2), respectively (log-rank test, *p* = 0.842). The 1-year survival proportion was 12.0% (95% CI: 0.0–24.7%) in the platinum group and 31.3% (95% CI: 8.4–54.2%) in the AMR group (Figure 2A). The median PFS in the platinum and AMR groups was 2.2 months (95% CI: 1.6–4.4) and 2.1 months (95% CI: 1.5–6.8), respectively (log-rank test, *p* = 0.405) (Figure 2B). No significant differences in OS or PFS were observed between the platinum and AMR groups. The ORR was 20.0% in the platinum group and 5.3% in the AMR group (OR 1.318 95% CI: 0.363–4.733, *p* = 0.213), whereas the DCR was 32.0% and 26.3%, respectively (OR 4.500 95% CI: 0.613–31.235, *p* = 0.682). No significant differences in ORR or DCR were observed between the two groups. Additionally, in the subgroup analysis according to the platinum-based regimen, there were no significant differences in OS between the platinum plus irinotecan and platinum plus etoposide groups (HR, 1.18; 95% CI, 0.49–2.83; *p* = 0.726). Similarly, no significant difference in PFS was observed between the two groups (HR, 1.12; 95% CI, 0.46–2.73; *p* = 0.808) (Supplementary Figure S1).

Next, a multivariate Cox proportional hazards analysis regarding prognosis was performed. First-line treatment response was defined as achieving a PR or better at the initial response assessment. None of the evaluated variables was significantly associated with OS. ECOG PS (HR, 1.110; 95% CI, 0.703–1.753; *p* = 0.654), clinical stage (HR, 2.446; 95% CI, 0.667–8.971; *p* = 0.177), first-line treatment response (HR, 1.025; 95% CI, 0.491–2.140; *p* = 0.947), and second-line treatment regimen (HR, 1.002; 95% CI, 0.514–1.955; *p* = 0.995) were not significantly associated with OS. Similarly, none of the evaluated variables was significantly associated with PFS. ECOG PS (HR, 1.337; 95% CI, 0.834–2.143; *p* = 0.228), clinical stage (HR, 2.254; 95% CI, 0.616–8.252; *p* = 0.220), first-line treatment response (HR, 0.678; 95% CI, 0.328–1.402; *p* = 0.294), and second-line treatment regimen (HR, 0.799; 95% CI, 0.411–1.551; *p* = 0.507) showed no significant association with PFS (Table 2).

**Figure 3:**
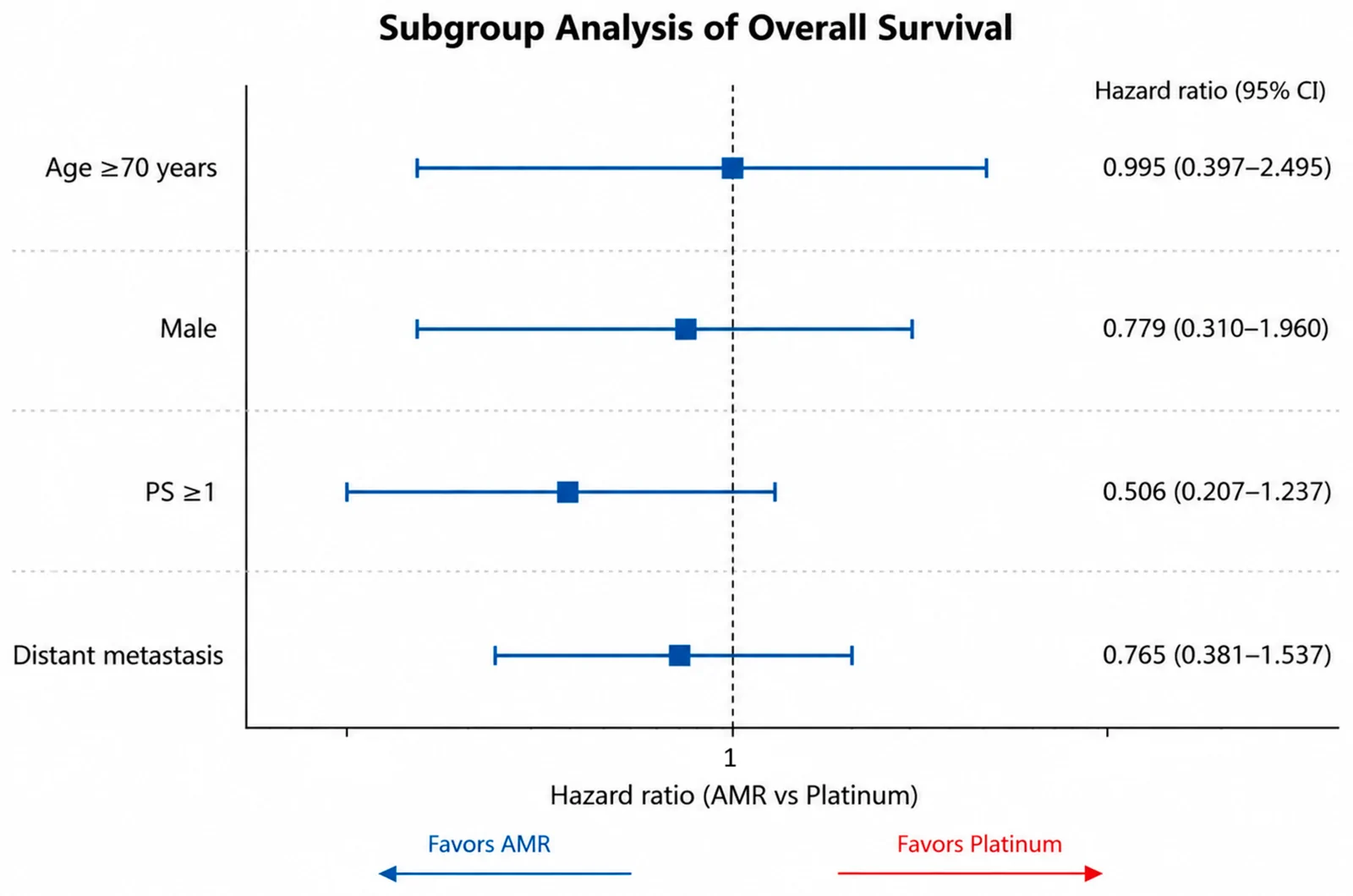
Subgroup analysis of overall survival. Forest plot showing hazard ratios (HRs) and 95% confidence intervals (CIs) for overall survival comparing amrubicin (AMR) with platinum-based chemotherapy across prespecified subgroups. The dashed line displays HR 1.0. An HR < 1.0 favors AMR, whereas an HR > 1.0 favors platinum-based chemotherapy.

Subgroup analyses of OS according to age, sex, performance status, and distant metastases showed no statistically significant differences between AMR and platinum-based chemotherapy (Figure 3). The HRs for AMR versus platinum-based chemotherapy were 0.995 (95% CI, 0.397–2.495) for age ≥ 70 years, 0.779 (95% CI, 0.310–1.960) for male sex, 0.506 (95% CI, 0.207–1.237) for PS ≥ 1, and 0.765 (95% CI, 0.381–1.537) for distant metastases. Although the point estimates tended to favor AMR in several subgroups, the confidence intervals were wide and crossed 1.0 in all subgroups.

Regarding treatment beyond the second line, 24 of the 44 patients (54.5%) received third-line treatment. The best response was stable disease in 3 patients, progressive disease in 17 patients, and unknown in 4 patients. The median overall survival from the initiation of third-line treatment was 3.7 months (IQR, 2.4–7.2 months).

### 3.3. AEs

Table 3 summarizes the AEs observed in the study population. The *p*-values presented reflect the results of the comparison and statistical testing of the frequency of Grade 3–4 events. Hematologic toxicities were the most common severe AEs in both treatment groups and were more frequent in the platinum group than in the AMR group. Grade 3–4 leukopenia and neutropenia were each observed in 15 patients (60.0%) in the platinum group, compared with five patients (26.3%) in the AMR group. Grade 3–4 thrombocytopenia occurred in six (24.0%) and three (15.8%) patients in the platinum and AMR groups, respectively. Grade 3–4 anemia was uncommon and was observed in only one patient (5.3%) in the AMR group. Non-hematologic grade 3–4 AEs were infrequent in both groups and included jaundice, fatigue, diarrhea, elevated alkaline phosphatase, liver dysfunction, and acute kidney injury. To evaluate the impact of primary prophylactic G-CSF on the incidence of grade 3–4 neutropenia, Fisher’s exact test was performed separately in the AMR and platinum groups. No significant association was observed between primary prophylactic G-CSF use and grade 3–4 neutropenia in either the AMR group (*p* = 0.583) or the platinum group (*p* = 1.000) (Supplementary Figure S2).

## 4. Discussion

In this multicenter retrospective study, AMR monotherapy and platinum-based doublet chemotherapy showed no statistically significant differences in clinical outcomes as second-line treatment for advanced EPNEC. Given the lack of an established standard of care beyond first-line platinum-based chemotherapy, these findings generate the hypothesis that amrubicin and platinum-based chemotherapy may have comparable efficacy as second-line treatment options, which warrants further prospective investigation.

Treatment selection after progression on first-line platinum-based therapy remains challenging in EPNEC [16]. Previous studies have suggested a survival benefit associated with chemotherapy in patients with EPNEC, with patients receiving chemotherapy showing significantly better survival than those who did not [3]. However, these studies primarily evaluated the overall impact of chemotherapy across different treatment settings and did not specifically address the optimal regimen after failure of first-line platinum-based therapy. In contrast, our study focused specifically on second-line treatment and directly compared amrubicin with platinum-based chemotherapy as treatment regimens. Although no statistically significant difference in OS or PFS was observed between the two regimens, our findings provide complementary evidence regarding the choice of second-line chemotherapy for EPNEC. Thus, whereas previous studies have primarily established the potential survival benefit of chemotherapy itself, our study addressed the clinically relevant question of which chemotherapy regimen may be appropriate after first-line platinum-based treatment. Similar difficulties have been reported in extensive-stage SCLC, which shares several clinicopathological and molecular characteristics with EPNEC. In SCLC, platinum rechallenge has demonstrated clinical benefit primarily in patients with platinum-sensitive relapse, whereas alternative agents such as AMR are generally favored in those with early disease progression [23,24,25]. Nonetheless, unlike SCLC, EPNEC lacks standardized definitions of platinum sensitivity and established treatment algorithms. Consequently, treatment decisions in EPNEC often rely on physician judgment and institutional experience rather than on robust evidence-based criteria.

While platinum-doublet chemotherapy may be preferred as second-line therapy in selected patients, repeated platinum exposure can be challenging for those who experienced cumulative toxicities during prior treatment. Conversely, irinotecan-containing regimens may not be suitable for patients with UGT1A1 risk genotypes because of their increased susceptibility to severe adverse events [26,27,28]. Several retrospective studies and clinical trials have reported the effectiveness of second-line treatments for EPNEC, particularly those using amrubicin or platinum-based regimens [13,14,29]. However, these studies largely focused on the efficacy of a single regimen, which makes it difficult to determine the relative value of different treatment strategies in routine clinical practice. Consequently, evidence directly comparing commonly used second-line regimens remains limited. Based on this study, clinicians can individualize treatment decisions according to prior treatment history, toxicity profiles, comorbidities, and patient preferences. This study addresses that evidence gap by directly comparing amrubicin and platinum-doublet chemotherapy in a real-world cohort of patients with EPNEC. To our knowledge, few studies have specifically evaluated these two treatment approaches within the same study population. Furthermore, the multicenter design represents a major strength of this analysis. Because EPNEC is a rare malignancy, most previous studies have been conducted at single institutions with relatively small sample sizes [30]. By incorporating data from multiple centers, this study better reflects contemporary clinical practice and enhances the generalizability of the findings. On the contrary, the characteristic of rapid disease progression in EPNEC may have contributed to systematically low estimates of ORR and DCR in our cohort. In some patients, disease progression or clinical deterioration may have occurred before radiological response assessment, resulting in classification as non-responders despite the absence of a formal post-treatment assessment. Thus, the low ORR and DCR should be interpreted with caution, as they may partly reflect the aggressive disease course rather than the intrinsic antitumor activity of the treatment. Additionally, the heterogeneity of primary tumor sites is another important consideration in interpreting our findings. EPNECs arising from different anatomical sites may differ in their biological characteristics, metastatic patterns, and treatment sensitivity, potentially influencing treatment selection and clinical outcomes. Previous studies have suggested that EPNECs arising from the genitourinary tract and head and neck may have relatively favorable outcomes compared with those arising from other sites [3]. However, these primary sites were relatively uncommon in our cohort. Therefore, the distribution of primary tumor sites may have contributed to the favorable survival outcomes observed in our study.

This study has some limitations. First, despite the cohort’s multicenter nature, its retrospective design may have introduced selection bias and unmeasured confounding factors. Although we observed no significant differences in baseline patient characteristics between the two treatment groups including response to first-line treatment as shown in Table 1 and, adjusted for several potential prognostic factors in the multivariate analysis, residual confounding cannot be excluded. In particular, unmeasured factors such as patient preferences, treatment goals, and other clinical considerations may have influenced treatment selection and subsequent outcomes. Second, pathologic diagnoses and radiologic response assessments were performed at each participating institution. Therefore, interobserver variability in pathologic classification and treatment response evaluation cannot be excluded. Third, adverse events were collected retrospectively from medical records, and low-grade toxicities (Grade 1–2) may have been underreported. Consequently, the safety profile presented here mainly reflects clinically significant adverse events. Finally, the incidence of hematologic toxicities may have been influenced by differences in G-CSF use among individual patients, potentially affecting the observed frequency of neutropenia. Fourthly, the limited sample size of 44 patients and 40 events resulted in a statistical power of only 24% to detect a true HR of 0.67, corresponding to a 33% reduction in risk, indicating that the study may have been underpowered to detect clinically meaningful differences. Nevertheless, this study has several important strengths. To our knowledge, it is one of the few multicenter studies directly comparing amrubicin monotherapy and platinum-doublet chemotherapy as second-line treatment options for patients with advanced EPNEC. Additionally, including multiple centers enhances the generalizability of our findings by reflecting real-world clinical practice across participating institutions. Given the rarity of EPNEC and the difficulty of conducting randomized clinical trials in this population, our findings may provide valuable real-world evidence for optimizing second-line treatment strategies.

## 5. Conclusions

AMR monotherapy and platinum-doublet chemotherapy showed no statistically significant differences in efficacy or safety as second-line treatments for advanced EPNEC. Prospective studies, including further investigation of predictive biomarkers and prognostic factors, are warranted to optimize treatment strategy. Treatment selection should remain individualized based on patient characteristics, prior treatment history, and clinical considerations until prospective comparative studies become available.

## Figures and Tables

**Table 1:** Baseline Characteristics among the patients with EPNEC.

No. (%)	Platinum (*n* = 25)	AMR (*n* = 19)	*p*-Value
Age, Median (IQR), years	70.0 (60.8–75.3)	65.0 (54.8–71.8)	0.225
Male	20 (80.0)	13 (68.4)	0.489
ECOG PS (0/1/2)	5/16/4	7/8/3	0.563
Second-line treatment cycles, Median (IQR)	2.0 (1.0–4.3)	3.0 (2.0–4.8)	0.129
First-line treatment response	10 (40.0)	6 (31.6)	0.565
Primary site			
Gastrointestinal tract			
Esophagus	3 (12.0)	2 (10.5)	1.000
Stomach	6 (24.0)	5 (26.3)	1.000
Duodenum	1 (4.0)	0 (0.0)	1.000
Cecum	1 (4.0)	0 (0.0)	1.000
Colon	1 (4.0)	1 (5.3)	1.000
Rectum	2 (8.0)	3 (15.8)	0.638
Anal canal	0 (0.0)	1 (5.3)	0.432
Hepatobiliary and pancreas			
Gallbladder	3 (12.0)	1 (5.3)	0.622
Pancreas	2 (8.0)	2 (10.5)	1.000
Others			
Prostate	1 (4.0)	1 (5.3)	1.000
Bladder	0 (0.0)	1 (5.3)	0.432
Uterus	1 (4.0)	0 (0.0)	1.000
Ovary	1 (4.0)	1 (5.3)	1.000
Parotid gland	1 (4.0)	0 (0.0)	1.000
Maxillary sinus	1 (4.0)	0 (0.0)	1.000
Unknown	1 (4.0)	1 (5.3)	1.000
Clinical stage			
Locally advanced	2 (8.0)	4 (21.1)	0.378
Metastases	23 (92.0)	15 (78.9)	
Histological feature			
Small cell carcinoma	11 (44.0)	6 (31.6)	
Large cell carcinoma	4 (16.0)	4 (21.1)	
NE	10 (40.0)	9 (47.4)	
Proliferative activity			
Ki-67 ≥ 50%	4 (16.0)	2 (10.5)	
Ki-67 < 50%	3 (12.0)	0 (0.0)	
NE	18 (72.0)	17 (89.5)	

Abbreviations: EPNEC, Extra-Pulmonary Neuroendocrine Carcinoma; AMR, Amrubicin; IQR, Interquartile Range; ECOG PS, Eastern Cooperative Oncology Group Performance Status; NE, Not Evaluated.

**Table 2:** Cox proportional hazards regression analysis for OS and PFS.

**Variable for OS**	**HR**	**95% CI**	***p*** **-** **Value**
ECOG PS	1.110	[0.703, 1.753]	0.654
Clinical stage	2.446	[0.667, 8.971]	0.177
First-line treatment response	1.025	[0.491, 2.140]	0.947
Second-line ETP-based regimen	1.002	[0.514, 1.955]	0.995
**Variable for PFS**	**HR**	**95% CI**	***p*** **-** **Value**
ECOG PS	1.337	[0.834, 2.143]	0.228
Clinical stage	2.254	[0.616, 8.252]	0.220
First-line treatment response	0.678	[0.328, 1.402]	0.294
Second-line ETP-based regimen	0.799	[0.411, 1.551]	0.507

Abbreviations: OS, Overall Survival; PFS, Progression-Free Survival; ECOG PS, Eastern Cooperative Oncology Group Performance Status; HR, Hazard ratio; CI, Confidence Interval; ETP, etoposide.

**Table 3:** Summary of Adverse Event Data According to CTCAE.

Adverse Event	No. (%)				
	Platinum (*n* = 25)		AMR (*n* = 19)		*p*-Value
	All Grades	Grade 3 and 4	All Grades	Grade 3 and 4	
White blood cell count decreased	22 (88.0)	15 (60.0)	9 (47.4)	5 (26.3)	0.026
Neutrophil count decreased	22 (88.0)	15 (60.0)	5 (26.3)	5 (26.3)	0.026
Anemia	13 (52.0)	0 (0.0)	7 (36.8)	1 (5.3)	0.432
Platelet count decreased	18 (72.0)	6 (24.0)	10 (52.6)	3 (15.8)	0.710
Jaundice	3 (12.0)	1 (4.0)	2 (10.5)	0 (0.0)	1.000
Fatigue	6 (24.0)	2 (8.0)	3 (15.8)	0 (0.0)	0.498
Diarrhea	2 (8.0)	1 (4.0)	1 (5.3)	0 (0.0)	1.000
Alkaline phosphatase increased	4 (16.0)	1 (4.0)	7 (36.8)	0 (0.0)	1.000
Liver dysfunction	5 (20.0)	0 (0.0)	3 (15.8)	1 (5.3)	0.432
Acute kidney injury	1 (4.0)	1 (4.0)	0 (0.0)	0 (0.0)	1.000
Febrile neutropenia		2 (8.0)		0 (0.0)	0.498

Abbreviations: CTCAE, Common Terminology Criteria for Adverse Events; AMR, Amrubicin.

## Data Availability

The datasets used and/or analyzed in the current study are available from the corresponding author upon reasonable request.

## Supplementary Materials

The following supporting information can be downloaded at: https://www.mdpi.com/article/10.3390/cancers18162664/s1, Figure S1: Kaplan–Meier curves for overall survival and progression–free survival according to second-line treatment. (A) Overall survival (OS) and (B) progression-free survival (PFS) in patients with advanced extrapulmonary neuroendocrine carcinoma (EPNEC) who received platinum + irinotecan or platinum + etoposide. Tick marks indicate censored observations. Figure S2: Association between primary prophylactic G-CSF use and grade 3–4 neutropenia. The association between primary prophylactic granulocyte colony-stimulating factor (G-CSF) use and the occurrence of grade 3–4 neutropenia was evaluated separately in the platinum-based chemotherapy and amrubicin (AMR) groups. Data are presented as *n* (%). *p*-values were calculated using Fisher’s exact test. G-CSF, granulocyte colony-stimulating factor; AMR, amrubicin.

## Abbreviations

AE	adverse event
AMR	amrubicin
AUC	area under the curve
CBDCA	carboplatin
CDDP	cisplatin
CD56	cluster of differentiation 56
CI	confidence interval
CR	complete response
DCR	disease control rate
ECOG	Eastern Cooperative Oncology Group
EPNEC	extrapulmonary neuroendocrine carcinoma
ETP	etoposide
G-CSF	granulocyte colony-stimulating factor
IQR	interquartile range
IRI	irinotecan
NEC	neuroendocrine carcinoma
ORR	objective response rate
OS	overall survival
PFS	progression-free survival
PR	partial response
PS	performance status
PTX	paclitaxel
SCLC	small-cell lung cancer
