# Supplementary material for: Clinical Outcomes of Second-Line Amrubicin and Platinum-Based Doublet Chemotherapy in Patients with Extrapulmonary Neuroendocrine Carcinoma: A Multicenter Retrospective Study
Source: Cancers (Basel). 2026 Aug 18;18(16):2664. doi: 10.3390/cancers18162664 (PMC13511585; doi:10.3390/cancers18162664)
Supplement: Supplementary file 1 [file cancers-18-02664-s001.zip › cancers-4462666-supplementary.pdf]

Supplementary Figure S1

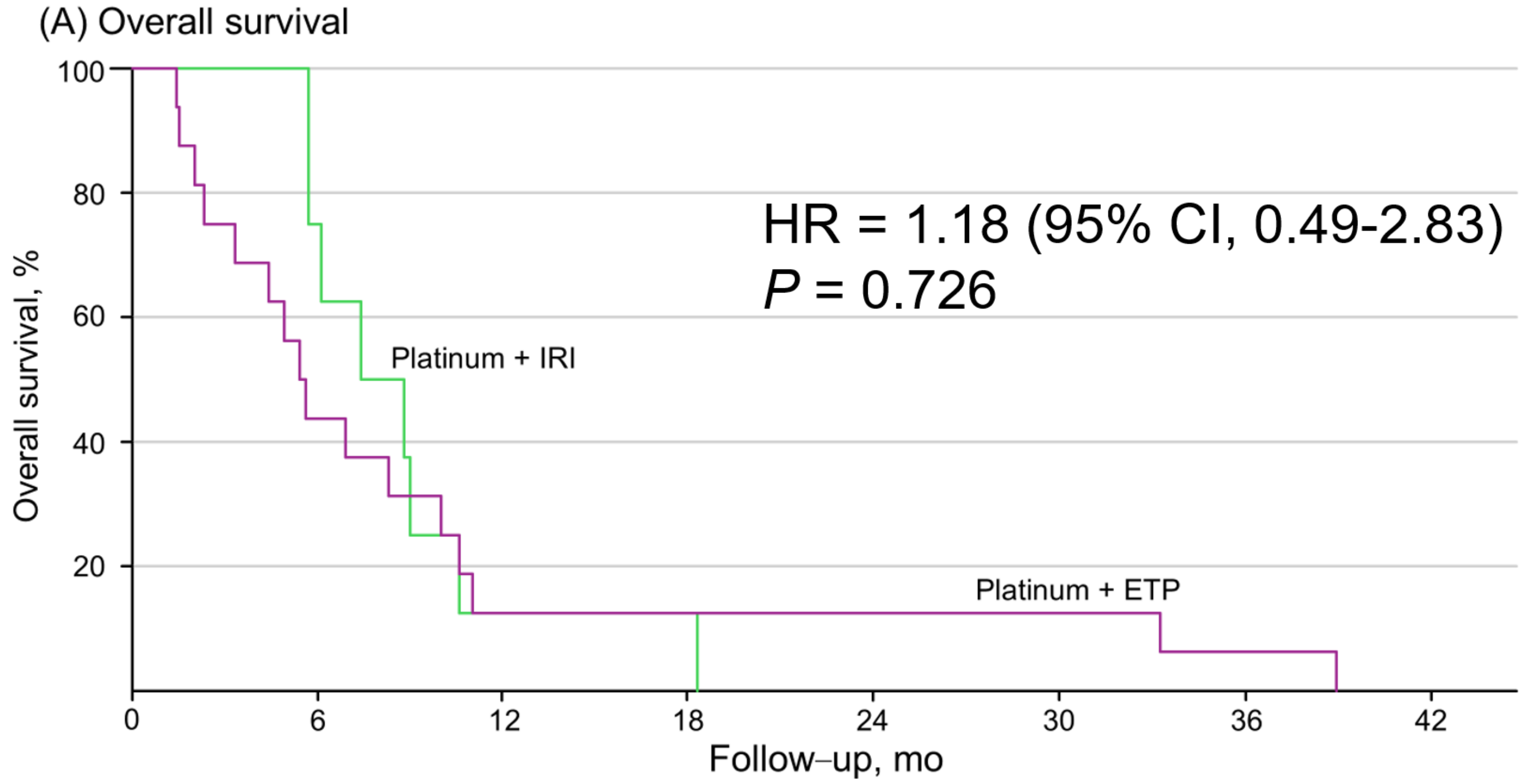

No. at risk (No. censored)

Platinum + IRI	8	7	2	2	0	0	0	0
Platinum + ETP	16	7	3	3	3	3	2	0

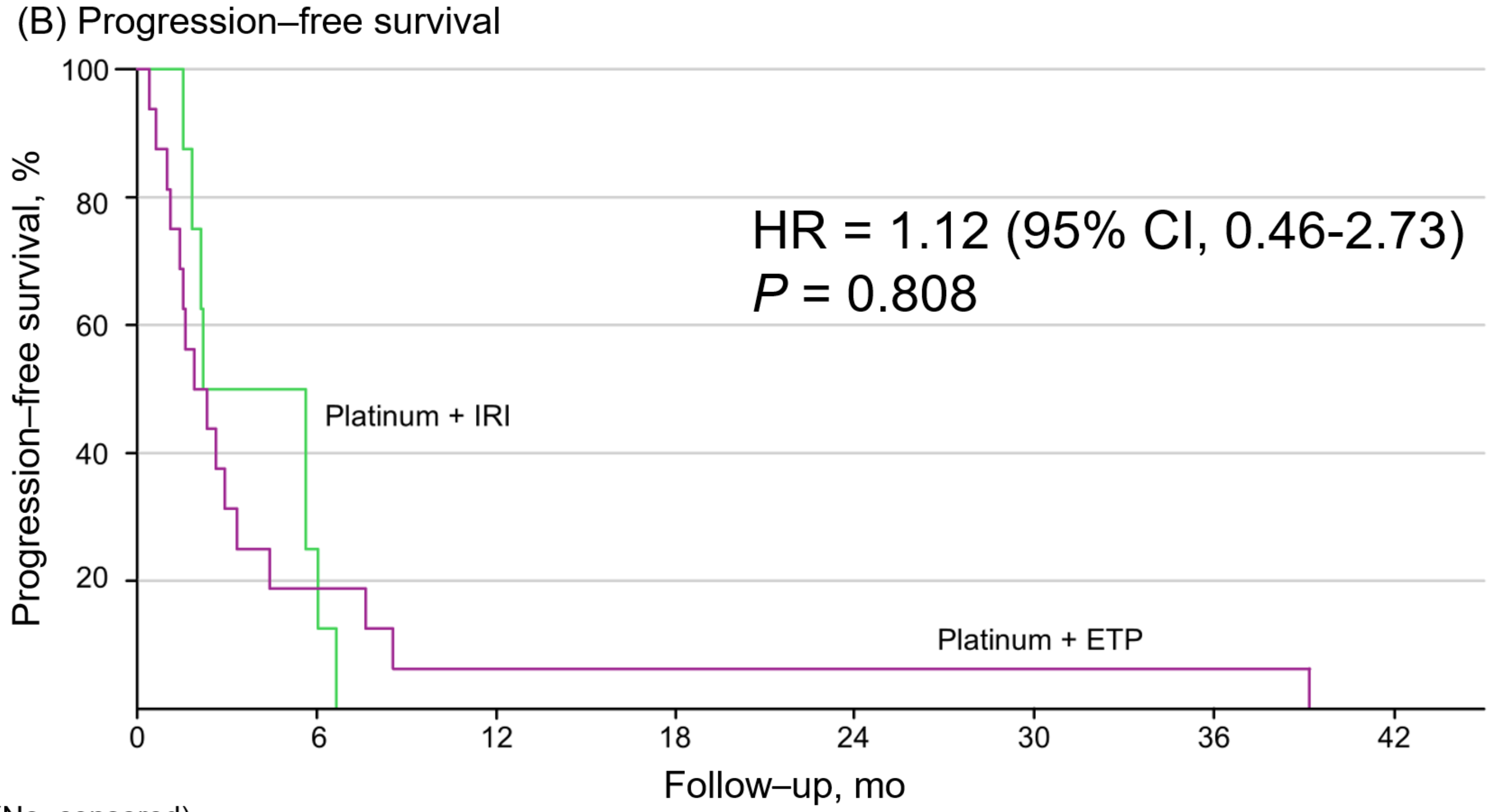

No. at risk (No. censored)

Platinum + IRI	8	2	0	0	0	0	0	0
Platinum + ETP	16	3	1	1	1	1	1	0

Supplementary Figure S1. Kaplan–Meier curves for overall survival and progression–free survival according to second-line treatment. (A) Overall survival (OS) and (B) progression-free survival (PFS) in patients with advanced extrapulmonary neuroendocrine carcinoma (EPNEC) who received platinum + irinotecan or platinum + etoposide. Tick marks indicate censored obser-vations.

Supplementary Figure S2

Platinum

		Neutrophil count decreased (Grade 3–4)	
		Yes<br>*n* (%)	No<br>*n* (%)
Primary prophylactic G–CSF	Yes	3 (30.0)	2 (20.0)
	No	12 (70.0)	8 (80.0)
P–value		1.000	

AMR

		Neutrophil count decreased (Grade 3–4)	
		Yes<br>*n* (%)	No<br>*n* (%)
Primary prophylactic G–CSF	Yes	3 (23.1)	10 (76.9)
	No	2 (40.0)	3 (60.0)
P–value		0.583	

Data are presented as *n* (%).

Supplementary Figure S2. Association between primary prophylactic G-CSF use and grade 3–4 neutropenia. The association between primary prophylactic granulocyte colony-stimulating factor (G-CSF) use and the occurrence of grade 3–4 neutropenia was evaluated separately in the platinum-based chemotherapy and amrubicin (AMR) groups. Data are presented as n (%). P-values were calculated using Fisher's exact test. G-CSF, granulocyte colony-stimulating factor; AMR, amrubicin.
